# Biome engineering‐2020

**DOI:** 10.1111/1751-7915.12391

**Published:** 2016-07-29

**Authors:** Harald Brüssow

**Affiliations:** ^1^ Nestlé Research CenterNutrition and Health ResearchHost‐Microbe InteractionVers‐chez‐les‐BlancCH‐1000Lausanne 26Switzerland

## Abstract

The gut microbiome research is going from a descriptive into an intervention phase. To optimize beneficial microbe–host interaction, we need to understand how to steer the system by modulating the nutrient input with which the system is literally fed (e.g. diets, fibres, prebiotics, human milk oligosaccharides), and we must learn how to modulate the composition of the gut microbiota by adding beneficial microbes (e.g. probiotics, faecal transplants) and by eliminating disturbing microbial members using, for example, bacteriophages in this highly complex ecosystem. The current status of the field is reviewed together with an outlook what might be expected until 2020, highlighting obstacles to progress and possible solutions to these problems.

In May 2016, The White House announced the launch of a National Microbiome Initiative to define the role of microbes in human and environmental health. On the heels of the NIH's Human Microbiome Project, this new initiative will likely lead to ‘biome engineering’ as a next hot topic in the microbiome field. A more applied outlook can also be predicted from numerous investments of industry into this research area which bets on a transition from descriptive to interventional microbiome initiatives. Also the European Union followed this trend with the ‘MyNewGut’ project where specific dietary intervention strategies are searched to modulate the gut microbiota for health benefits. In addition, the Gates Foundation launched a project exploring bacteriophages as tools for biome engineering. The present perspective explores the potential and prospect for microbiota modulation. In view of the large literature, it concentrates on human data from the gut microbiome and is focused on its biotechnological feasibility, not on health benefit aspects.

## The gut biome: complexity complicates interventions

**Figure 1:**
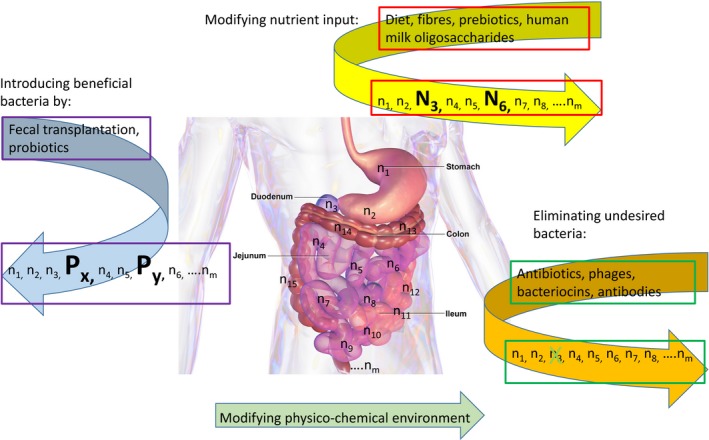
Schematic representation of three main ways to manipulate the gut microbiome investigated in the present perspective. The intestinal microbiota is represented by its members n_1_, n_2_ … n_m_ along the length of the gut projected onto the human body. By changing the nutrient intake (diet change, prebiotics, fibres and human milk oligosaccharides), the growth of specific microbes can be stimulated as indicated by the two members of the gut consortium displayed with large capital letters. By faecal microbiota transplantation and oral probiotics application, beneficial microbes can be introduced into the gut which were lost from the system indicated by the large members P_x_ and P_y_. Finally, with antibiotics, bacteriophages, bacteriocins and specific oral antibodies, undesired members of the gut microbiota can be eliminated as indicated by the crossed member of the microbiota in case of a bacteriophage application. In case of antibiotics, a more pervasive elimination or reduction of gut microbes would be expected. A final possibility, not further discussed in this article, is by modification of the physico‐chemical environment of the gut as for example by drug application. The background gut picture is from Blausen.com staff *Wikiversity Journal of Medicine*.

In the past, physiologists and microbial ecologists have described the gut as an anaerobic bioreactor and have developed models consisting of a series of glass vessels mimicking the different gut segments. Even in this simplified model, complexity is conferred by the large number and diversity of microbes which contribute a vast number of genes which surpass those of the host by two orders of magnitudes. Gut bacteria provide not only complementary metabolic pathways for energy harvest to the host but also assist in food digestion, detoxification and the production of bioactive compounds. In such a bioreactor, the task of microbial biotechnologists is clear: to optimize the output of the system for the host, we need to understand how to steer the system (i) by modulating the nutrient input with which the system is literally fed; and we must learn how to modulate the composition of the microbial part of the bioreactor (ii) by adding beneficial and (iii) by eliminating disturbing microbial members of this bioreactor. A further although non‐physiological possibility is (iv) to change the physico‐chemical condition of the niche (Fig. 1). It will certainly be of value to study the impact of common oral medication (e.g. antacids, proton pump inhibitors) on the composition of the gut microbiota, but so far data have mostly been obtained on the biotransformation of drugs by the gut microbiota and the impact of antibiotics on the gut microbiota.

The goal for ‘Biome engineering‐2020’ is thus easily set, but as usual, it is easier said than done. With the feed input, we touch one of the most diverse and within a specific ethnic group also most conservative habits of humans – traditional food and eating with all its cultural connotations. With respect to the members of this bioreactor, we are dealing with trillions of microorganisms representing between 100 and 1000 distinct bacterial species. Substantial progress has been achieved in the description of the gut microbiota by high throughput 16S rRNA gene and metagenome sequencing. With greater technical possibilities, the complexity of the task has increased. We can expect that with metagenome and 18S rRNA gene sequencing, the virome and the eukaryotic part of the gut microbiome, respectively, will also come into focus. As some of them feed on bacteria, their interaction with the gut microbiota might merit ecological considerations. Another technical breakthrough will be instrumental for gut microbiota research over the next years. For a long period, many gut microbes led a purely *in silico* existence. Bacterium–bacterium and bacterium–host interactions were deduced from sophisticated bioinformatics network analyses. Now the Sanger Center after years of systematic bacterial genome sequencing reported the culturing of 137 bacterial species from the stool of six healthy adults, many of them were new bacterial species; even new bacterial families were isolated (Browne *et al*., 2016). What was until recently considered an ‘unculturable’ part of the human gut microbiota is now accessible for phenotypic analysis and became tools for intervention studies.

## Another layer of complexity: the gut biome as a new human organ

Over the last decade, the perception of the gut has experienced a substantial paradigm shift. The gut acquired physiological roles that go well beyond classical characteristics of a bioreactor. The ensemble of the gut microbes were lifted to the status of a ‘new organ’ of the human body which plays crucial roles for human physiology (Brown and Hazen, 2015). Considering microbes as an ‘alter ego’ of the human condition is philosophically an appealing idea suggesting another Copernican revolution in human self‐perception which might explain the great interest of the public in microbiota research and the hype not only of the lay press but also in the scientific literature announcing new discoveries in the microbiome field. Some researchers see the microbiome as the result of an ancient bacterial‐animal co‐evolution deducing from this statement many beneficial interactions. However, microbes will fill every habitat on earth which offers nutrients and appropriate physico‐chemical conditions for microbial growth and the gut is certainly not an exception. We should, therefore, be more cautious in anticipating a mutually profitable relationship between gut microbes and the host without further experimental proof. Some microbes have certainly co‐evolved with humans (as pathogens show not to our benefit), while other bacteria have evolved with respect to adaptation to external conditions and following pressures from microbe–microbe interactions.

A number of microbe–host interactions have been demonstrated over the last decade: among other things, gut microbes were postulated to play crucial roles in human obesity and malnutrition (Blanton *et al*., 2016), which is not implausible since nutrient uptake is the primary task of the gut. During early colonization of the gut, microbes were recognized as instructors for the maturation of the immune system. Commensals were postulated to provide a colonization resistance barrier against infection and invasion with gut pathogens. With the extension of the role of the gut microbiota as a new organ in the human body, new challenges confront the biotechnologist who is interested in biome engineering since interventions should not only change the output of the bioreactor but also further human health.

## A better definition of biome dysbiosis

In the current literature, there is a trend to associate an equilibrated gut microbiota with human health and a deviation from an equilibrium (‘dysbiosis’) with disease. The list of diseases associated with altered gut microbiota composition is rapidly growing and ranges from allergy to irritable bowel syndrome, from cancer to metabolic syndrome, from inflammatory bowel disease to diabetes, reaching to mood and psychological disorders. Numerous ‘axes’ were defined that link the gut with the lung, the skin and the brain and the gut microbiota is frequently considered as an important transducer of effects. Disturbances in the gut microbiota were, for example, reported to affect via a ‘gut–brain axis’ blood glucose homeostasis (De Vadder *et al*., 2014) and even behavioural conditions like autism (Hsiao *et al*., 2013).

However, the field is fraught with several difficulties. First, a major limitation for a deeper analysis is the inaccessibility of the gut. In most publications, the gut is still treated as a black box where only the food or bacterial input into the mouth and the bacterial output in the stool are analysed and intervening processes occurring along a length of more than 8 metres in humans are only indirectly inferred, with most evidence coming from rodents. Some light into the darkness of the hidden parts of the human gut is provided by surgical resections, rare fistula patients or well‐motivated medical students accepting invasive catheters and smart pills. Second, most gut microbiota‐disease connections are still at the level of associations, it is not yet clear whether the altered microbiota is the cause or the consequence of the disease. Third, the dysbiosis of the gut microbiota is frequently not clearly defined with respect to increases and decreases, losses and acquisitions of specific members of the gut microbiota defined at a finer taxonomical level. The diagnosis of an altered ratio of *Firmicutes* and *Bacteroidetes* phyla, for example, in obesity is not sufficiently detailed to guide specific interventions by microbial biotechnologists with defined bacterial strains, unless whole foetal microbiota transplantation is considered. In addition, many gut microbiota‐disease associations are only described at a sophisticated, but highly abstract level which leaves the microbial biotechnologist wondering how to translate principal components into interventions. Fourth, many studies were performed with very few subjects. In view of the tremendous compositional diversity between individuals where each individual's microbiota is considered by some researchers as unique as a fingerprint, one might ask how reliable these associations are, particularly when knowing that the microbiota composition of a ‘healthy’ human population is influenced by many factors. These confounding factors must be controlled before association studies can lead into intervention trials.

## Obstacles and solutions

To overcome these difficulties, a few approaches are necessary. For example, as done in clinical sciences, we need meta‐analyses for major gut microbiota‐diseases associations based on studies by different groups and in different populations, with studies fulfilling certain methodological criteria on which we can then base decisions to promote or inhibit specific bacteria in targeted biome interventions. To attract grants, researchers are tempted to probe into new microbiota‐disease associations which lead to papers in high impact journals which prefer to publish new and exciting reports. Yet, for public health is equally important to repeat studies, which is scientifically less awarding for authors. There is currently a broad discussion about a reproducibility crisis in scientific research. One might, therefore, encourage grant agencies to reserve money to re‐investigate gut microbiota‐disease associations in different populations for topics of public health importance.

In addition, we need a definition what represents a healthy reference gut microbiota. In view of the intensive sequencing efforts of the Human Microbiome Project, this task looks deceptively simple – but it is not. Infants, children, adults and elderly have a distinct gut microbiota. Even in a single age group, the gut microbiota shows substantial diversity. For example the ELDERMET study described proportions for *Bacteroidetes* to *Firmicutes* ranging from 0.03 to 0.94. When plotted for individuals, a continuous distribution was obtained for the *Bacteroidetes/Firmicutes* ratio (Claesson *et al*., 2011). How do we define then a normal reference gut microbiota? Are tabulations of gut microbiota compositions needed for the major microbial groups in different age ranges, in different geographical regions and for distinct life styles? Only when we know the breadth of gut microbiota distribution in apparently healthy subjects, we can estimate to what extent a group of patients with a given disease deviates significantly from that ‘normal’ distribution. This task might not be so easy since it is conceivable that different degrees of ‘healthiness’ might exist which show a gradual transition into a disease state (Brüssow, 2013a) – the ELDERMET study is a good example for this concept. Furthermore, certain diseases in gut microbiome analyses might represent not a uniform clinical entity, but several distinct clinical conditions currently covered by a single name (e.g. obesity, inflammatory bowel disease, irritable bowel syndrome) which could blur clear associations with better defined clinical conditions.

Finally, a certain trend for overinterpretation of experimental results is apparent in the microbiome field. I will illustrate the dilemma by a highly quoted, influential paper (De Filippo *et al*., 2010). Far‐reaching conclusions on the influence of diet were drawn from a gut microbiota comparison of African and Italian children, where the children clearly differ in many other potentially confounding factors in addition to diet composition (amount of calories, antibiotic use, length of breastfeeding, climate and environmental hygiene). In fact, a similar north–south gradient was also seen in children from Europe: children from Spain differed significantly from those of UK or Germany for *Bifidobacterium, Bacteroides* and *Enterobacteriaceae* despite displaying a similar breastfeeding rate (Fallani *et al*., 2010). As in classical case–control and epidemiological studies, we need matched local controls who differ only in a single, to be investigated trait from the cases. Interesting conjectures and hypotheses are giving colour to research and appeal for the larger public, but they would best be formulated in opinion papers. An important role is here for reviewers who need to look critically on conclusions that eventually go well beyond the presented experiments.

## Biome engineering: modifying the nutrient input

### The vegetarian diet

The most obvious intervention for gut biome engineering is by modifying the food intake (Brüssow and Parkinson, 2014; Graf *et al*., 2015). This can be done by switching from one to another type of diet or more subtly by supplementing a defined chemical compound that feeds specific bacterial groups (‘prebiotics’). The seminal paper for the first approach was published by David *et al*. (2014). These authors offered 10 US adults a plant‐based or an animal‐based diet for 4 days. These short‐term consumptions of distinct diets changed the microbial composition in the stool in a specific way. The animal‐based diet increased abundance of bile‐tolerant microbes (*Alistipes putredinis*,* Bilophila* and *Bacteroides*), which are associated with amino acid fermentation. The plant‐based diet showed a correlation with saccharolytic microbes (*Roseburia*,* Eubacterium rectale*,* Ruminococcus bromii*,* Faecalibacterium prausnitzii*) associated with carbohydrate fermentation. One feels immediately reminded of Metchnikoff's ideas about the benefits of a saccharolytic over a putrefactive gut microbial metabolism expressed in his influential 1907 book ‘*The Prolongation of Life: Optimistic Studies’* where he linked the consumption of yogurt and *Lactobacillus* with longevity in humans.

Contrary to the short‐term effects described by David *et al*. (2014), no consistent microbiota pattern emerged from studies with long‐term differences in diet. Omnivorous compared with vegetarian young Indian women displayed a weakly increased proportion of *E. rectale* (Kabeerdoss *et al*., 2012). Germans on vegetarian or vegan diet showed a modest decrease in *Bacteroides*,* Bifidobacterium* and *Escherichia coli* compared with matched omnivores (Zimmer *et al*., 2012). Vegetarians from Slovenia demonstrated a higher faecal concentration of the *Bacteroides‐Prevotella* group than omnivores (Matijašić *et al*., 2014). A small group of US vegetarians showed a non‐significant increase in *Prevotella* enterotype (Wu *et al*., 2011). Why are the effects so small? One reason could be that the effect of the diet changes on the microbiota did not overcome the inter‐subject variations in the intestinal microbiota (Walker *et al*., 2011; Wu *et al*., 2011). Notably, only studies using a cross‐over design where both diets were given to the same subjects showed clear effects (David *et al*., 2014). Another reason could be that the orally ingested food represents only part of the food source for the gut microbiota. Together with the ingested food, an adult secretes daily about 7–8 l of fluid into the gut containing many biomolecules to which must be added a continuous release of mucins and a desquamation of enteric epithelial cells which together represents a convenient and constant nutrient source for many gut bacteria.

The Dutch LifeLines‐DEEP study investigated the faecal microbiome from 1135 participants with metagenome sequencing and analysed microbiome associations with 126 intrinsic and exogenous factors (Zhernakova *et al*., 2016). Only 19% of the variation seen in the inter‐individual distance of microbial composition is explained by these 126 factors; we are thus far from an understanding what determines the gut microbiome composition. From the dietary side, the study associated consumption of coffee, tea and red wine (which all have a high polyphenol content), but not a vegetarian diet with increased diversity of the faecal microbiome. The consumption of buttermilk, a fermented dairy product, in contrast to whole milk drinking led to a higher faecal content of dairy bacteria, suggesting some potential to ‘probiotic’ drinks. In the future, we might see reports that screen chemical fractions from food ingredients identified in such epidemiological studies for growth modulation of defined gut microbes either by in vitro test or in small invertebrates (worms, insects) to guide human intervention trials in the future.

### Intervention trials with dietary fibres

Some dietary interventions with defined chemical compounds were already conducted in humans. Feeding resistant starch as native granules or as retrograded starch led to increased faecal titres of *E. rectale* and *R. bromii* (Abell *et al*., 2008; Martínez *et al*., 2010; Walker *et al*., 2011), while chemically modified starch led to increases in *Actinobacteria* and *Bacteroidetes*. Plant‐derived dietary fibres [inulin, fructo‐oligosaccharides (FOS)] or fibres produced by enzymatic synthesis [galacto‐oligosaccharides (GOS)] led in nutritional intervention trials to a substantial increase of *Bifidobacterium*. For a mixture of FOS/GOS, researchers could document not only a 10‐fold faecal bifidobacterial titre increase over placebo recipients (maltodextrin) but also beneficial clinical effects. From meta‐analysis, there is some evidence that a prebiotic supplement added to infant feeds may prevent eczema (Osborn and Sinn, 2013). FOS and GOS also led in adults to an increase in faecal *Bifidobacterium* with slightly beneficial laxative effects (Kleessen *et al*., 1997) and on cholesterol excretion.

### Human milk oligosaccharides

A fascinating addition to the field of microbiota‐modulating compounds is human milk oligosaccharides (HMOs). HMO is of no energetic value for the infant since it resists enzymatic hydrolysis by host enzymes (Engfer *et al*., 2000). This seemingly wasteful biosynthesis represents a paradox. In one hypothesis, HMOs serve as receptor decoys for gut pathogens (Morrow *et al*., 2004). An alternative hypothesis considers HMO as nutrients for commensal gut microbes. *Bifidobacterium* and *Bacteroides* species could metabolize HMO, but not enteric *Streptococcus*,* Veillonella*,* Eubacterium*,* Clostridium* and *E. coli* assuring a selectivity of HMO as growth promoter for specific bacteria (Marcobal *et al*., 2010). Considerable differences in HMO utilization exist among infant bifidobacteria opening opportunities for fine tuning with HMO (Matsuki *et al*., 2016). Different *Bifidobacterium* or *Bacteroides* isolates followed different enzymatic strategies of either intracellular or extracellular HMO digestion. The latter strategy allows also bystander bacteria to exploit the liberated sugars (Rakoff‐Nahoum *et al*., 2016) with possibilities of cross‐feeding of enteric pathogens (Ng *et al*., 2013). Some bifidobacteria showed preferences for fucosylated or small mass HMO typically produced through the first month of lactation (LoCascio *et al*., 2007). The pioneers in that emerging field even suggested that mothers use a ‘glycan code’ when synthesizing HMO during the breastfeeding period that ‘instruct’ the gut in their infants what particular bacteria should get a nutritional push during a given time interval. As decoys for the multitude of gut pathogens and as feed for so many gut symbionts the bewildering diversity of HMO with about 200 described HMO chemical species would then suddenly make sense in the light of evolution (Varki, 2006). If these ideas are verified, an enormous biotechnological potential for a species‐ or even strain‐specific stimulation of gut bacteria would open up with HMO. Several safety and tolerance trials with HMO were already registered in healthy infants or adults by diverse food and biotech companies.

## Introducing beneficial bacteria into the gut microbiome

### Faecal microbiota transplantation (FMT)

Recurrent *Clostridium difficile* infection (CDI) is difficult to treat. In a seminal study, a small number of these patients were treated with a high dose of vancomycin, bowel lavage and faeces from healthy donors given by a nasoduodenal tube (van Nood *et al*., 2013). The effect of faecal transplantation was striking: 81% of the patients were cured after the first faecal infusion compared with a cure rate of 23% and 31% in two control groups. *Bacteroidetes*,* Clostridium* clusters IV and XIVa which were low in the patients increased after treatment to the levels displayed by the donors and bacilli which were high in the patients decreased substantially. *Clostridium difficile* decreased from initial 4% to 0.2% after FMT. The low microbiota diversity in the patients increased to the diversity of the donors within 2 weeks (van Nood *et al*., 2013; Fuentes *et al*., 2014). A recent trial reproduced an 84% cure rate with faecal transplantation given as enema to 232 Canadian CDI patients, who were after intensive antibiotic pretreatment randomized to either fresh or frozen stool preparations, without difference in cure rate (Lee *et al*., 2016). The frozen material represents major advantages with respect to stool provision and in cost reduction with donor screening for enteric pathogens.

Shahinas *et al*. (2012) reported success of FMT in only half of the investigated CDI patients where *Proteobacteria* (*Escherichia*) were replaced by four *Bacteroidetes* species. Seekatz *et al*. (2014) associated FMT with a decrease in *Proteobacteria* (48% in CDI: mostly *Cronobacter* and *Enterobacteriaceae*) and an increase in *Bacteroidetes* (1% in CDI). Shankar *et al*. (2014) observed high amounts of *Gammaproteobacteria* and Bacilli in CDI patients (41% and 34% respectively) which decreased to 2% upon FMT with a concomitant increase in Clostridia (76%). Interestingly, *C. difficile* was not detected in the patients before FMT. Hamilton *et al*. (2013) described a shift from *Proteobacteria* to *Firmicutes* and *Bacteroidetes* with FMT, but a patient who needed subsequently antibiotics for a urinary infection returned to a dominance of *Escherichia*. Weingarden *et al*. (2015) found the same overall shift from *Proteobacteria* to *Bacteroidetes* and *Firmicutes* after FMT. Directly after transfer, donor and recipients were highly correlated for faecal microbiota, but diverged substantially over later time points. Millan *et al*. (2016) reported that patients were dominated by *Proteobacteria*, mostly *Escherichia* and *Klebsiella*. FMT led to their replacement by *Bacteroidetes* and *Firmicutes*. This community shift was also accompanied by a decrease in number and diversity of antibiotic resistance genes suggesting that high frequency of faecal *Enterobacteriaceae* in CDI patients might reflect selection by the intensive antibiotic treatment schedules. The beneficial effects of FMT in CDI were, thus, well reproduced; less well established are the long‐term effects of FMT and the persistence of the beneficial effects.

### Future trends in FMT

The microbiological but not the clinical effect of FMT has now also been tested in metabolic syndrome patients (Li *et al*., 2016). The proof of bacterial transfer from donor to recipient was done at strain level by single‐nucleotide variant analysis which demonstrated a variable introduction pattern of donor strains. In ulcerative colitis patients, FMT showed no clinical benefit in a controlled trial (Rossen *et al*., 2015a). For other gut conditions the conclusions are not yet clear (Rossen *et al*. 2015b). Standardization of faecal material is obviously a regulatory problem (Vyas *et al*., 2015); pathogens can be transmitted with donor stools, but also multiple lineages of temperate phages, which potentially carry virulence genes and can destabilize the microbiome in recipients (Antonopoulos and Chang, 2016; Chehoud *et al*., 2016).

Faecal microbiota transplantation is not a new technique and did not depend on recent technological advances (de Vos, 2013). The first successful application of FMT in CDI was described by US physicians in 1958 (Eiseman *et al*., 1958). In the late 1980s, Danish researchers developed a cocktail consisting of 10 faecal commensal bacterial species and demonstrated their clinical efficacy in CDI patients (Tvede and Rask‐Madsen, 1989). By cultivation microbiology, these researchers could associate *Bacteroides* (specifically *B. ovatus, B. vulgatus* and *B. thetaiotaomicron*) with clinical recovery. Since *C. difficile* inhibited *Bacteroides* in vitro, the researchers suspected that a prior application of vancomycin against *C. difficile* is necessary to allow the outgrowth of the grafted *Bacteroides* strains which then inhibited the re‐growth of *C. difficile* (Tvede and Rask‐Madsen, 1989). Positive clinical effects in CDI were recently also documented along this line with synthetic microbiomes consisting of 33 defined strains (Petrof *et al*., 2013) or an even larger, undefined set of gut commensals propagated in vitro (Jorup‐Rönström *et al*., 2012). A compromise between diversity of transferred microbes, which is for many researchers the basis for the clinical efficacy of FMT in CDI, and biological safety is a spore preparation representing approximately 50 species of spore‐forming *Firmicutes* derived from seven healthy donors. The stool was treated with 50% ethanol which eliminates vegetative pathogens, fungi, viruses while retaining all spore formers. In an open, single arm trial with 30 recurrent CDI patients treated with these spores, no recurrence was observed for 87% of the patients which compares very favourably with historical controls. The gut microbiota was remodelled by the spores: *Bacteroides* titres increased and *Klebsiella* carriage decreased (Khanna *et al*., 2016). Another group worked with spores from a single non‐toxigenic *C. difficile* strain M3 based on the hypothesis that this strain is best equipped to compete with toxigenic *C. difficile* for a niche in CDI patients. In a placebo‐controlled multicentre trial with 173 CDI patients, the spores reduced recurrence from 30% to 11%. Recurrence occurred essentially in those treated patients were the spores failed to colonize the patients (Gerding *et al*., 2015).

### Probiotics

Mixed results were obtained on gut microbiota composition with oral application of probiotic bacteria. To quote some examples: In healthy adults, *Lactobacillus paracasei* DG induced an increase in *Proteobacteria* and a decrease in the Clostridiales genus *Blautia*; both returned to their initial state after cross‐over to placebo (Ferrario *et al*., 2014). Different commercial preparations containing either probiotic or dairy *Bifidobacterium*,* Lactobacillus*,* Lactococcus* or *Streptococcus* strains caused no significant changes in the overall structure of adult gut microbiota (Kim *et al*., 2013) or on the functional profile of faecal microbiome genes (McNulty *et al*., 2011). Intervention with *Lactobacillus rhamnosus* LGG in adults increased during the supplementation period the faecal excretion of the probiotic, but had no measurable effect on the composition of the gut microbiota (Lahti *et al*., 2013). A fermented milk containing *Lactobacillus acidophilus* and *B. animalis* was given to adults with irritable bowel syndrome together with dietary fibre in a placebo‐controlled trial (Matijašic *et al*., 2016). Again, the global profile of the faecal microbiota was not altered except for a transient increase in the two probiotic strains during supplementation. In adults, *L. rhamnosus* combined with a weight reduction diet had no overall impact on faecal microbiota composition and weight loss, while subgroup analysis showed an effect in women (Sanchez *et al*., 2014). Overall, the trials showed a great resilience of the microbiota in adults towards probiotic supplementation. Even if the microbiota profile is the same, we cannot exclude that the function of the microbiota is affected by probiotic supplementation.

The situation is different in infants where the gut microbiota is still in a maturation phase. Supplementation of infants with *L. rhamnosus* LGG showed in comparison with placebo a shift in stool community composition with an increase in Lactobacillaceae and Bifidobacteriaceae (Cox *et al*., 2010). The effect was confirmed in preschool children who showed a 5‐fold increase in *Lactococcus* and *Lactobacillus* and a 3‐fold decrease in *E. coli* (Korpela *et al*., 2016b). Children born by caesarean section (CS) showed a different early gut microbiota colonization compared with vaginally delivered (VD) infants. CS infants were randomized < 3 days after birth to receive *Lactobacillus reuteri* or control formula. Supplemented, but not control CS infants showed at 2 and 16 weeks of age a gut microbiota composition approaching that of VD infants (Rodenas *et al*., 2016). The effect was more prominent than a vaginal microbiota transplantation trial which recently made headlines (Dominguez‐Bello *et al*., 2016).

## Eliminating undesired bacteria from the gut microbiome

### Antibiotics

The impact of oral antibiotics on bacterial pathogens has been extensively studied to achieve an optimal coverage of bacterial pathogens commonly associated with a given infection type. It has long been suspected that antibiotics also cause lateral damage on the gut microbiome, and that the loss of obligate anaerobes results in decreased colonization resistance against new infections (*Salmonella*). Antibiotic use is frequently associated with an expansion of γ‐*Proteobacteria* and enterococci (Pamer, 2016). Antibiotic‐associated diarrhoea is a frequent clinical observation. Based on these observations, it was proposed to identify commensal gut bacteria that can be developed into next‐generation probiotics to re‐establish colonization resistance after or together with antibiotic treatment (Pamer, 2016). However, the impact of antibiotics on the gut biome was studied by 16S rRNA gene sequencing only since relatively recently and we are still far from possessing a detailed knowledge of what type of antibiotic causes what specific effect on the commensal gut microbiota. Such data are needed to screen for antibiotics that hit the pathogen, but not so much the commensals which are responsible for colonization resistance. Indeed, a prospective study in Finnish children showed that macrolide antibiotics caused a long‐lasting shift in microbiota composition and metabolism, while penicillins left a much weaker mark (Korpela *et al*., 2016a). The above‐mentioned gut microbiome dysbiosis in CDI is a lively reminder of the extent of biome disturbance by an intense antibiotic treatment schedule and its clinical consequences. Initial concepts that compared antibiotic treatment with the devastating effect of herbicides on a flowering meadow do, however, not reflect the reality. Many antibiotic effects are more subtle (as shown in another study with Finnish children, Yassour *et al*., 2016), vary from chemical class to chemical class of antibiotics and from individual to individual, and reflect preceding antibiotic treatments. A study by Dethlefsen and Relman (2011) illustrates the situation. Three adults who each experienced two courses of treatment with ciprofloxacin were followed with 50 samples over 10 months. Day‐to‐day temporal variability was evident and inter‐individual variation was the major source of variability between the samples. Against this natural fluctuation, the effect of ciprofloxacin was profound and rapid: the researchers observed a shift in community composition and a loss of diversity. However, 1 week after the treatment, the communities began to return to the initial state, while substantial differences were seen between the three subjects. Loss and gain of specific community members were not identified, but shifts around equilibrium positions occurred demonstrating a great resilience and functional redundancy of the gut microbiota.

Pérez‐Cobas *et al*. (2013a) described distinct microbiota shifts in four patients treated with either bacteriostatic or bacteriocidal antibiotics. Again, inter‐individual variability was the greatest factor of difference such that subject‐specific responses will complicate the analysis of antibiotic class‐specific effects on the microbiota. Also paradoxical short‐term effects were reported: 21 patients treated with fluoroquinolones or β‐lactams showed as expected a 25% microbial diversity decrease and a reduction of the core microbiota by more than half of the taxa, but instead of a decrease, a slight increase in bacterial load was described (Panda *et al*., 2014). The current dilemma of the field is well illustrated by another article by Pérez‐Cobas *et al*. (2013b) where a single patient treated with intravenous β‐lactam was analysed by a multi‐omic approach. A complex microbial response was observed displaying oscillatory population dynamics which raises doubts whether antibiotics might become usable tools for the microbial biotechnologist for targeted microbiome modulation until 2020.

### Bacteriophages

Gut metagenome analyses identified the virome as an essential part of the gut microbiome. Since bacterial viruses (‘bacteriophages’) represent the greatest share of the gut virome and since lytic phages only survive via infection and lysis of their bacterial target cells, phages need to be integrated into a comprehensive description if we want to understand the ecological functioning of the gut microbiome. It will be interesting to see whether concepts developed by marine microbial ecologists like ‘killing the winning population’ (Wommack and Colwell, 2000) also apply to the gut or whether alternative concepts putting more emphasis on temperate phages are more relevant for the gut (Knowles *et al*., 2016). For the next years, interesting insights can be expected from this field which will introduce new dynamic and mechanistic aspects into a gut microbiota research and its modulation.

In 2015, The American Academy of Microbiology issued a report where the authors recommended the use of microbes as therapeutics (http://academy.asm.org/index.php/browse-all-reports/5296-harnessing-the-power-of-microbes-as-therapeutics-bugs-as-drugs). One section of this report deals with bacteriophages for treating bacterial infections. The authors mention many assets of phage therapy (PT): speed and specificity of lytic action; a self‐titrating dose; activity against biofilms; no safety issues when restricted to lytic phages. These aspects are supported by a wealth of in vitro data and treatment experiments in animal infection models. Particularly, in view of their species‐specific lytic action, phages appear also as ideal tools for manipulating the gut microbiome, more specifically for eliminating only a single targeted bacterial species. Two aspects have so far hampered further progress with this otherwise promising approach. PT has been investigated in animal models demonstrating a low impact on the non‐targeted gut microbiota (for a recent example, see Galtier *et al*., 2016). Yet translating these results into clinical application is a large step since the understanding of phage–bacterial interaction in the human host is still in its infancy (Brüssow, 2013a, 2016). PT is in Eastern Europe a registered drug and is used for treating a wide range of bacterial infections with different phage cocktails. However, controlled clinical trials are, with one notable exception from the 1960s, largely lacking for the eastern European phage preparations (Vandenheuvel *et al*., 2015). Only one proof of concept clinical trial exists for an ear infection in the Western literature (Wright *et al*., 2009). A recent PT treatment trial of *E. coli* diarrhoea in children from Bangladesh did not result in clinical amelioration over standard therapy (Sarker *et al*., 2016). Despite gut transit of viable phage, phages had no impact on *E. coli* or the gut microbiome. However, *E. coli* was only present with low titres and was not correlated with clinical symptoms while a marked dysbiosis with faecal streptococci was observed in the acute phase of diarrhoea patients. While PT is concept‐wise an attractive option for biome engineering, a clear clinical proof of efficacy must still be provided. However, the spectre of antibiotic‐resistant bacteria urges further exploration of phages as potential novel anti‐microbial agents. Bacteriophages are not the only tool for a targeted microbiome manipulation. Bacteriocins and specific antibodies are further alternatives. A hyperimmune bovine colostrum showed in a recent animal trial treatment effects against CDI without affecting the gut microbiota composition (Sponseller *et al*., 2015).

## Outlook

We are currently seeing gut microbiota research in a transition phase from a descriptive into an interventional phase. Until 2020, substantial progress can be anticipated for microbiome correction in CDI patients. For microbiome modulation in young children, industrial products might soon enter a development phase. For other areas, the complexity and intrinsic resilience of the adult gut microbiome together with its high inter‐individual variability makes progress until 2020 a difficult task. It might, therefore, be advisable to develop concepts of biome engineering in less complex and more accessible human microbiota (nares, nasopharynx, vagina, skin) and to return with that knowledge to the task of targeted manipulation of the gut microbiome. In these simpler human ecosystems, it will also be easier to explore microbiome manipulation with finer tools like regulatory RNAs, quorum sensing compounds, metabolic cross‐feeding, designer probiotics and synthetic consortia than in the gut system.

## Conflict of interest

None declared.
